# Tumor-infiltrating lymphocytes in breast cancer predict the response to chemotherapy and survival outcome: A meta-analysis

**DOI:** 10.18632/oncotarget.9988

**Published:** 2016-06-13

**Authors:** Ke Wang, Jianjun Xu, Tao Zhang, Dan Xue

**Affiliations:** ^1^ Department of Oncology, Second Affiliated Hospital, Zhejiang University School of Medicine, Hangzhou, 310009, China; ^2^ School of Finance, Zhejiang University of Finance and Economics, Hangzhou, 310018, China; ^3^ Department of Plastic Surgery, Second Affiliated Hospital, Zhejiang University School of Medicine, Hangzhou, 310009, China

**Keywords:** breast cancer, lymphocyte infiltrates, pathological complete response (pCR), prognosis, meta-analysis

## Abstract

Tumor-infiltrating lymphocytes (TILs) influence tumor prognosis and the chemotherapeutic response. Here, we quantified the clinical relevance of TILs, including the effect of TILs on lymphocyte subpopulations and assessed their consistency in breast cancer. We searched published literature from January 2000 to January 2016. The main parameters analyzed were pathological complete response (pCR) and survival outcome following chemotherapy in patients with breast cancer. Pooled odds ratio (OR) or relative risk (RR) values with 95% confidence intervals (CIs) were computed using random and fixed-effects models. Subgroup and heterogeneity analyses were also conducted. Twenty-three studies, which included 13,100 patients, met the inclusion criteria. The pooled results showed that TILs were associated with clinicopathological parameters of biologically aggressive phenotypes, such as high tumor grade or estrogen/progesterone receptor negativity, but they were not correlated with human epidermal growth factor receptor-2 expression. Moreover, a high TIL level was associated with a significantly improved pCR rate compared with a low TIL level (OR, 2.81; *P* < 0.001), particularly in the triple-negative breast cancer subtype (OR, 4.67; *P* < 0.001). An analysis of lymphocyte subpopulations showed that infiltration by CD8 lymphocytes, but not by CD4 lymphocytes and Foxp3 cells, was associated with a high pCR rate. Furthermore, a high TIL level was associated with significantly longer disease-free survival and overall survival. Our present meta-analysis indicates that an increased number of TILs predicted pCR to chemotherapy and improved survival. A high TIL level, characterized mainly by the infiltration of CD8 lymphocytes, is a strong predictive and prognostic factor.

## INTRODUCTION

Increasing evidence indicates that the interplay between the immune system and cancer cells is critical for tumor progression [1, 2]. However, the interactions between immune cells and cancer are amazingly complex. Although the results of previous studies examining the relationship between tumor-infiltrating lymphocytes (TILs) and breast cancer are conflicting, recent results have indicated an association between lymphocytic infiltration and improved neoadjuvant chemotherapy response and a favorable prognosis [3, 4].

Traditional thought has been that chemotherapy is be immunosuppressive; however, a new concept has emerged that cell death (apoptosis) triggers an immune system response, and certain types of chemotherapies may enhance cytotoxic lymphocyte responses and confer permanent anti-tumor immunity [5]. In particular, chemotherapy may result in cell death and releases tumor antigens, which processed by antigen-presenting cells and activating tumor-specific CD8+ T-cells. The ability to induce cell death might be critical for long-lasting tumor remission. In this way, conventional cytotoxic chemotherapy serves as a form of immunotherapy. Therefore, the pretreatment immune status may predict the ability of chemotherapy to eliminate cancer cells.

The prognostic value of breast cancer TILs was originally demonstrated in BIG 02–98 trial [6]. Loi et al. suggested that a 10 percent TILs increment in stromal was associated with an 18 percent reduction in the risk of death in patients treated with doxorubicin-based chemotherapy. Subsequently, many studies on the role of the immune system in breast cancer development and outcomes were reported, especially in the setting of TNBC and HER2-positive breast cancer. However, a pathological clinical evaluation of TIL levels did not recommend as a new prognostic factor in breast cancer patients at the 2015 St. Gallen Consensus Conference, because there are no standardized guidelines for clinical validation and its relevance is not completely understood.

Current opinions on the features of TILs and their prognostic potential in breast cancer are inconsistent. A meta-analysis of TILs and adjuvant chemotherapy found only a modest protective effect of TILs and did not include data regarding long-term survival outcomes [7]. Moreover, emerging data linking TIL expression to outcomes in large clinical trials do not include studies from 2015–2016. Thus, there is a need for updated information. Many recent larger studies have focused mainly on TILs that clinically respond to anthracycline- or taxinol-based therapy, in which patients were categorized by TIL group [8–11]. These studies might help us further understand breast cancer and explain the clinical usefulness of TILs as predictive markers. In this study, we identified the association between TILs and short-term clinical response to therapy and survival in patients with breast cancer.

## RESULTS

### Description of the studies

**Figure 1:**
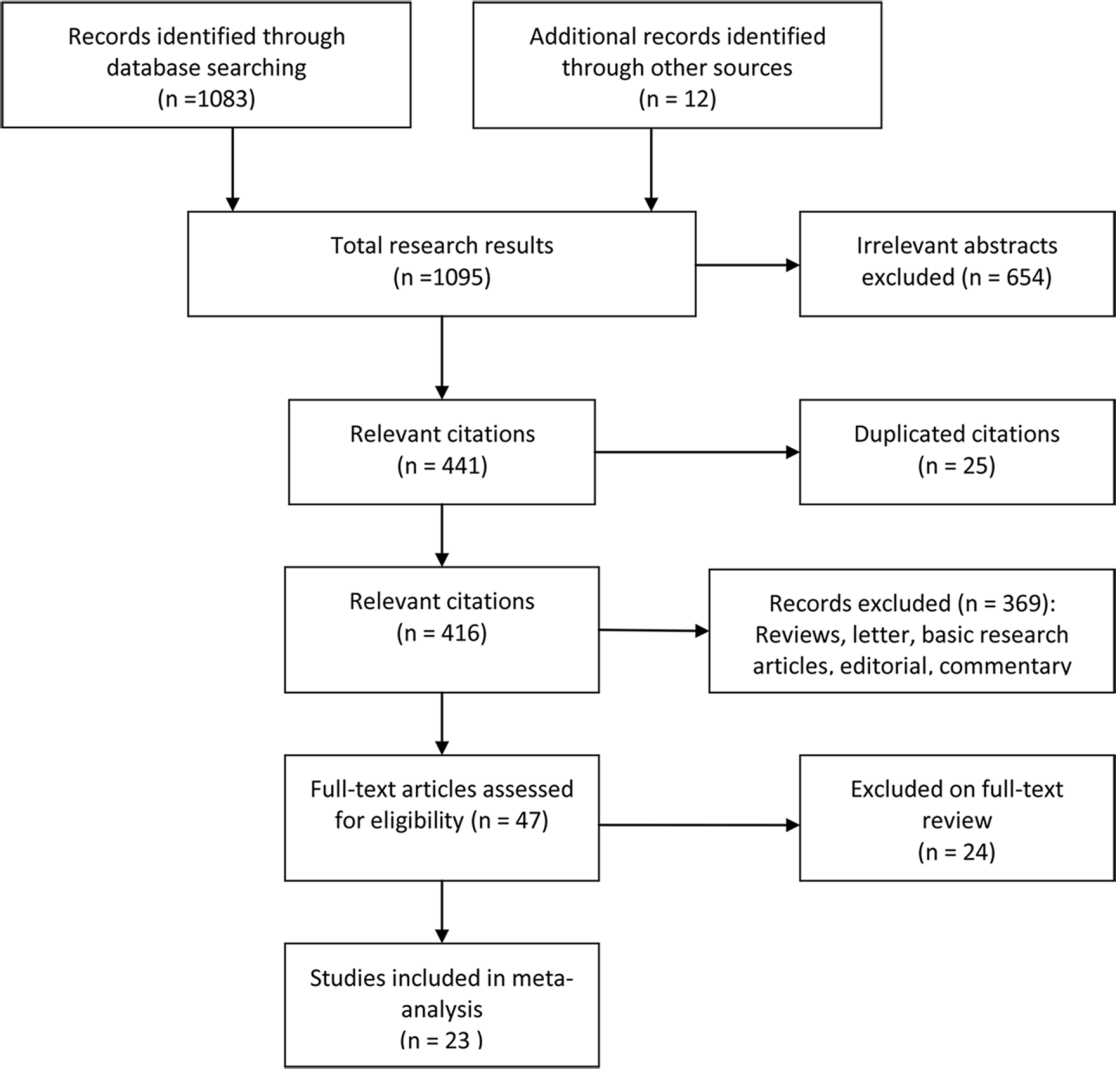
Flow chart outlining the process of study selection

A total of 1,095 records were identified in a primary literature search (Figure 1). We excluded 654 studies, including laboratory studies or irrelevant studies after screening, based on the inclusion criteria. Another 394 studies were excluded because they were reviews, had too small sample size, were duplicate articles, or provided no survival outcome data. Twenty-four articles were excluded because they lacked essential results concerning data extraction. After excluding studies, 23 studies met the criteria for evaluation, comprising 13,100 cases [6, 8–29]. Although references 20 and 27 came from the same authors, the studies evaluated the distinct outcomes of pathological complete response (pCR) and disease-free survival (DFS). Each cohort ranged from 60 to 2,596 patients and was published between 2009 and 2015. The main characteristics of the studies included are summarized in Table 1.

**Table 1:** Clinical characteristics of the eligible studies

Study	Country	Year	Tech	No. of patients	TILs phenotype	Cut-off value	pCR	Chemotherapy	Outcome	Median follow up
**Aruga**	Japan	2009	IHC	87	FOXP3	Median	Japanese criteria	CEF, EC	pCR, RFS, OS	3.9 years
**De Kruijf**	Netherlands	2010	IHC	677	FOXP3	Presence	NR	NR	OS	19 years
**West**	Canada	2011	IHC	368	CD3, CD4, CD8, FOXP3	Fisher's exact test	ypT0	A	pCR, DFS	NR
**DeNardo**	Missouri	2011	IHC	179	CD4, CD8, CD68	NR	NR	PTX	OS	4.3 years
**Ono**	Japan	2012	H&E	180	TILs	TILs > 50%,	ypT0	A/T, FEC	pCR, DFS	5.4 years
**Oda**	Japan	2012	IHC	180	CD8, FOXP3	Median	ypT0	CEF,T	pCR	NR
**Liu**	China	2012	IHC	132	FOXP3	Median	ypT0	FEC, CEX	PFS,OS	5.2 years
**Yamaguchi**	Japan	2012	H&E	64	TILs	Median	ypT0	A/T	NR	NR
**Loi**	Belgium	2013	H&E	2016	TILs	TILs ≥ 50%	ypT0	A/T, Tra	DFS, OS	8 years
**Seo**	Korea	2013	IHC	153	CD4, CD8, FOXP3	Median	ypT0	A	pCR	NR
**Lee**	Korea	2013	IHC	175	TILs	TILs ≥ 40%	ypT0	A/T, Tra	pCR	NR
**Issa- Nummer**	Germany	2013	H&E	313	TILs	TILs ≥ 60%	ypT0	EC-T,	pCR	NR
**Nabholtz**	France	2014	IHC	60	CD8	≥ 118 member/field	ypT0	A/T,	pCR	NR
**Adams**	USA	2014	H&E	506	TILs	TILs ≥ 50%	ypT0	A/T	DFS, OS	10.6 years
**Miyashita**	Japan	2014	IHC	110	CD8, FOXP3	Median	ypT0	NAC	pCR	NR
**Gaicía- Martínez**	Spain	2014	IHC	121	CD3, CD4, CD8, CD68	Median	ypT0	A/T	pCR, DFS,OS	5 years
**Lee**	Korea	2015	IHC	447	TILs	TILs > 60%	NR	A/T, Tra	DFS	4.1 years
**Salgado**	Australia	2015	IHC	455	TILs	TILs ≥ 12.5%	ypT0	CT, Tra	pCR, EFS	3.8 years
**Engels**	Netherlands	2015	IHC	2596	HLAs, FOXP3	FOXP3 > 49 positive cells	NR	CT	RFP, OS	5 years
**Dieci**	France	2015	H&E	781	TILs	TILs > 50%	NR	A/T	OS	12.7 years
**Denkert**	USA	2015	H&E	580	TILs	TILs ≥ 60%	ypT0	A/T, P, Tra	pCR,	NR
**Pruneri**	Italy	2015	H&E	897	TILs	TILs ≥ 50%	NR	A/T	DFS, OS	8.2 years
**Perez**	USA	2015	IHC	2027	TILs	TILs > 60%	NR	A/P, Tra	DFS	4.4 years

Abbreviations Tech, technique; pCR, pathological complete response; IHC, immunohistochemistry; H&E staining, hematoxylin-eosin staining; TILs, tumor-infiltrating lymphocytes; FOXP3, transcription factor forkhead box protein3; CT, chemotherapy; NAC, neoadjuvant chemotherapy; A, anthracycline-based chemotherapy; A/T, anthracycline- or taxane- based chemotherapy; P, paclitaxel; FEC, fluorouracil/epirubicin/cyclophosphamide; EC-T, epirubicin/cyclophosphamide-docetaxel; Tra, trastuzumab, DFS, disease-free survival; PFS, progression-free survival; OS, overall survival; NR, not reported.

### Correlations between TILs and clinicopathological parameters

**Figure 2:**
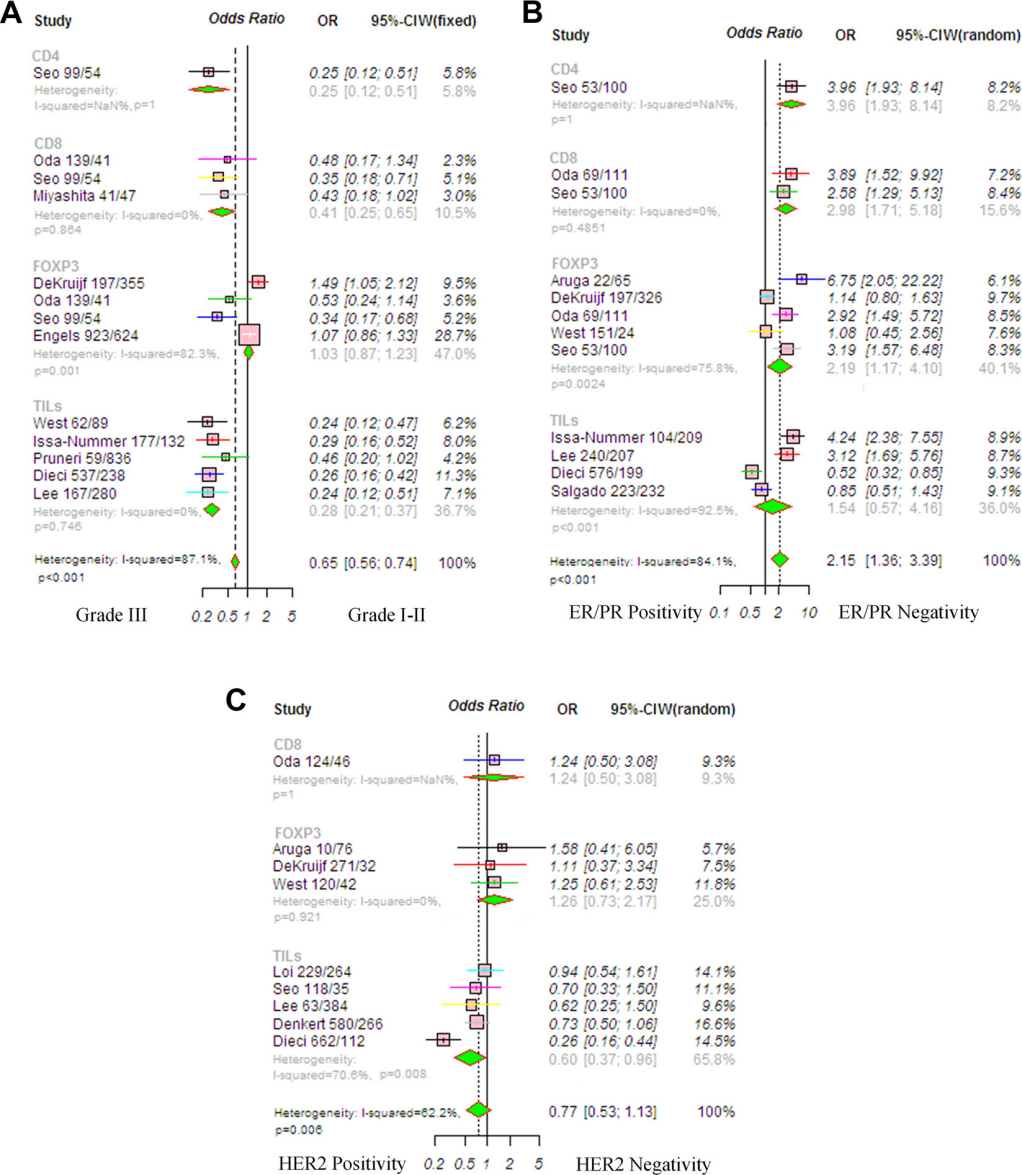
Forrest plot of odds ratio (OR) used to assess the correlations between tumor-infiltrating lymphocyte (TIL) status and clinicopathological features (**A**) Tumor grade, random-effects analysis; (**B**) ER/PR status, random-effects analysis; (**C**) HER2 status. Horizontal lines indicate 95% confidence intervals (CIs), and squares represent the OR of each individual study. Diamonds represent the overall OR, and the solid vertical line was set at the null value (OR 1.0). Abbreviations: TNBC, triple negative breast cancer; HER2, human epithelial growth factor receptor 2; ER, estrogen receptor.

Ten studies were available that examined the relationship between TILs and clinical parameters, such as tumor grade (Figure 2A). Because the heterogeneity among studies was significant (*P* < 0.001, *I*^2^ = 87.1%), the random-effects model was applied. The risk estimate with a pooled OR was 0.45 (95% CI, 0.30–0.68, *P* < 0.001), suggesting that TILs are associated with a high histological grade. The trim-and-fill analysis revealed that seven studies might be missing and that, if these studies were published, the adjusted OR would be 0.42 (95% CI, 0.27–0.65, *P* < 0.001, random-effects model). Nine articles investigated TILs and estrogen receptor/progesterone receptor (ER/PR) status (Figure 2B), with a pooled OR of 2.15 (95% CI, 1.36–3.39, *P* = 0.001). Because the heterogeneity among studies was significant (*P* < 0.001, *I*^2^ = 84.1%), the random-effects model was applied. The trim-and-fill analysis revealed that four studies might be missing, and if these were published, the adjusted OR would be 2.14 (95% CI, 1.35–3.36, *P* = 0.001, random-effects model). This result indicates that TILs are correlated with ER/PR negativity. However, the pooled data show that TILs were not associated with human epidermal growth factor receptor 2 (HER2) expression (OR, 0.77; 95% CI, 0.53–1.13, *P* = 0.181; Figure 2C). Because the heterogeneity among studies was significant, (*P* = 0.001, *I*^2^ = 62.2%), the random-effects model was applied. The trim-and-fill analysis revealed that three studies might be missing and that, if these were published, the adjusted OR would be 0.72 (95% CI, 0.58–1.04, *P* = 0.06, random-effects model).

### Impact of TILs on pCR in patients with breast cancer

**Figure 3:**
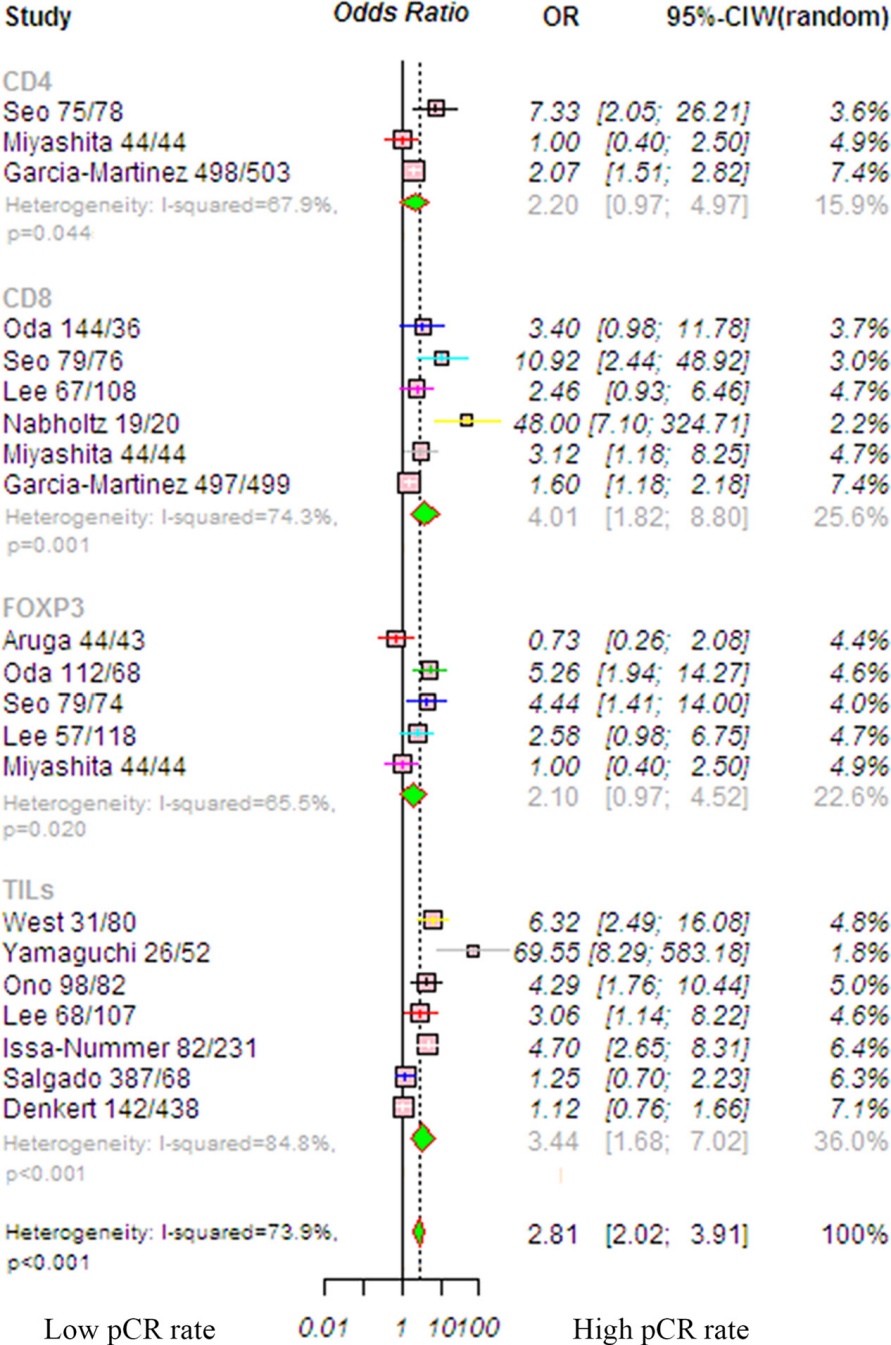
Forrest plot of OR used to assess the correlations between TIL status and pCR for the overall population OR values with 95% confidence intervals for response to chemotherapy are associated with high versus low TILs; an OR > 1.0 indicates that a patient with a high TIL level has a better pCR rate compared to that with a low TIL level. Abbreviations: TILs, tumor-infiltrating lymphocyte; TNBC, triple-negative breast cancer; HER2, human epithelial growth factor receptor 2.

The usefulness of TILs in the clinical response to therapy was identified using the pooled OR method. We analyzed the impact of each lymphocyte subpopulation in the entire group and in subgroups to determine their contribution. Because the heterogeneity among studies was significant (*P* < 0.001, *I*^2^ = 73.9%), the random-effects model was applied. The results showed that the pCR rate for patients with breast cancer was significantly higher for patients in the high TIL group than those in the low TIL group (OR, 2.81; 95% CI, 2.02–3.91, *P* < 0.001; Figure 3). Most differences were found with CD8 lymphocytes, which are cytotoxic T cells and have been linked to a better pCR rate (OR, 4.01; 95% CI, 1.82–8.80, *P* < 0.001). The CD4 lymphocyte subgroup (OR, 2.20; 95% CI, 0.97–4.97, *P* = 0.057) and the Foxp3 subgroup (OR, 2.10; 95% CI, 0.97–4.52, *P* = 0.058) had no predictive value. Trim-and-fill analysis revealed that eight studies might be missing and that if these were published, the adjusted OR would be 1.74 (95% CI, 1.23–2.47, *P* = 0.002, random-effects model). A meta-regression was conducted with the following covariates: markers, detection method, TIL cut-off, and chemotherapy. The results showed that no variable significantly influenced the OR estimate (Supplementary Table S1).

**Figure 4:**
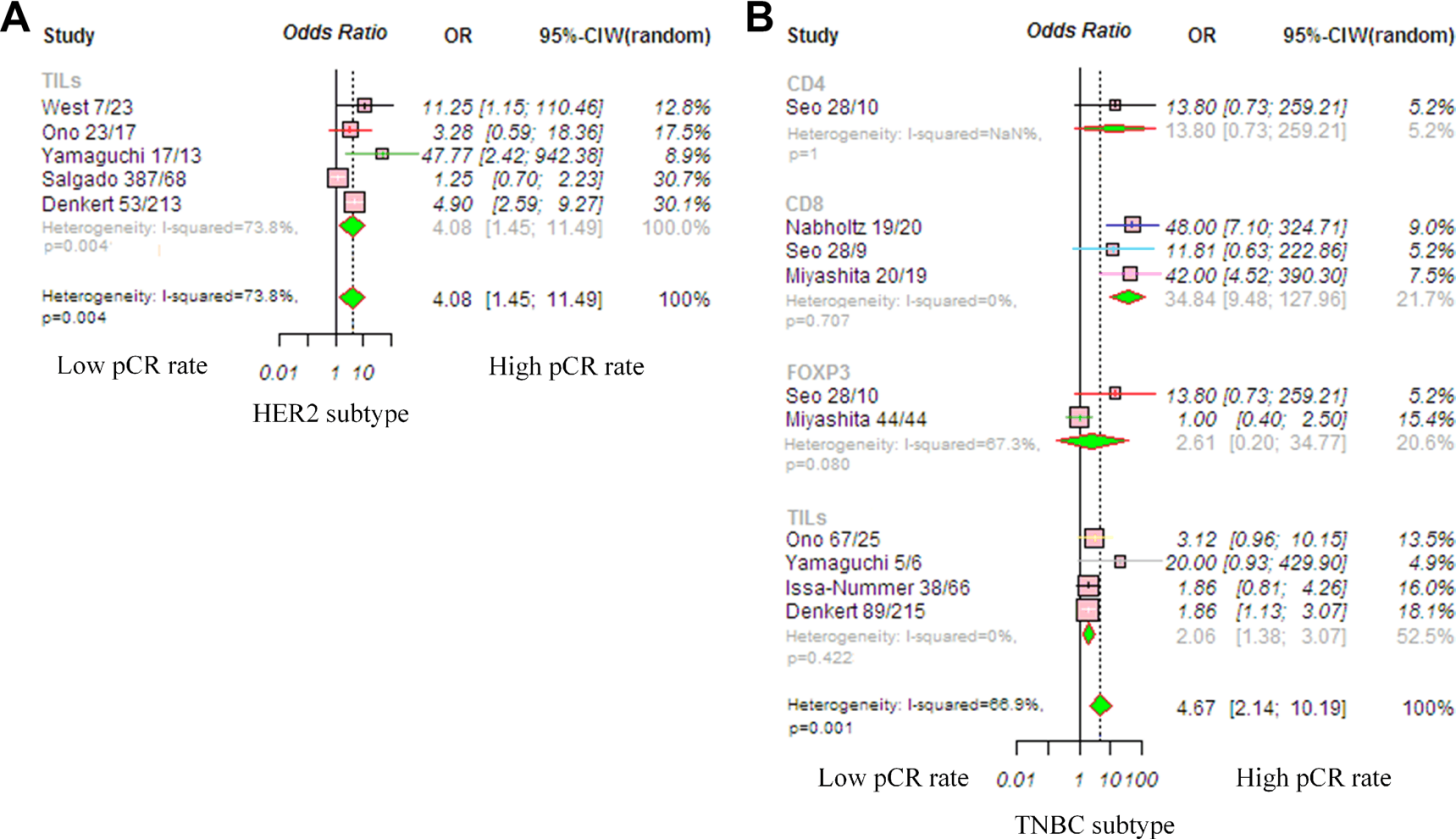
Forrest plot of odds ratio (OR) used to assess the correlations between TIL status and breast cancer subtypes (**A**) HER2-positive disease; (**B**) TNBC disease. OR values with 95% confidence intervals for response to chemotherapy are associated with high versus low TILs; an OR > 1.0 indicates that a patient with a high TIL level has a better pCR rate compared to that with a low TIL level. Abbreviations: TILs, tumor-infiltrating lymphocyte; TNBC, triple-negative breast cancer; HER2, human epithelial growth factor receptor 2.

A pooled subgroup analysis on the pCR was performed in HER2-positive patients. Five studies were available to analyze the association between TIL status and pCR. Because the heterogeneity among studies was significant (*P* = 0.001, *I*^2^ = 73.8%), we used the random-effects model, and the pooled OR of the overall effect was 4.08 (95% CI, 1.45–11.45, *P* < 0.004; Figure 4A). This was consistent with previous studies, revealing that a high TIL level significantly increased the pCR rate. The trim-and-fill analysis revealed that two studies might be missing and that if these were published, the adjusted OR would be 3.72 (95% CI, 1.41–9.83, *P* = 0.008, random-effects model).

The pooled OR for TNBC found an increased pCR in patients with high levels of TILs (OR, 4.67; 95% CI, 2.14–10.19, *P* < 0.001; Figure 4B); the random-effects model was used because the heterogeneity among studies was significant (*P* = 0.001, *I*^2^ = 66.9%). The predictive value of TILs for pCR was significant in the CD8 lymphocyte subgroup (OR, 34.84; 95% CI, 9.48–127.96, *P* < 0.001), but not significant in the CD4 (OR, 13.80; 95% CI, 0.73–259.21, *P* = 0.079) or Foxp3 subgroups (OR, 2.61; 95% CI, 0.20–34.77, *P* = 0.266). The trim-and-fill analysis revealed that four studies might be missing and that if these were published, the adjusted OR would be 4.67 (95% CI, 2.14–10.18, *P* = 0.008, random-effects model).

### Effects of TILs on disease free survival (DFS) in patients with breast cancer

**Figure 5:**
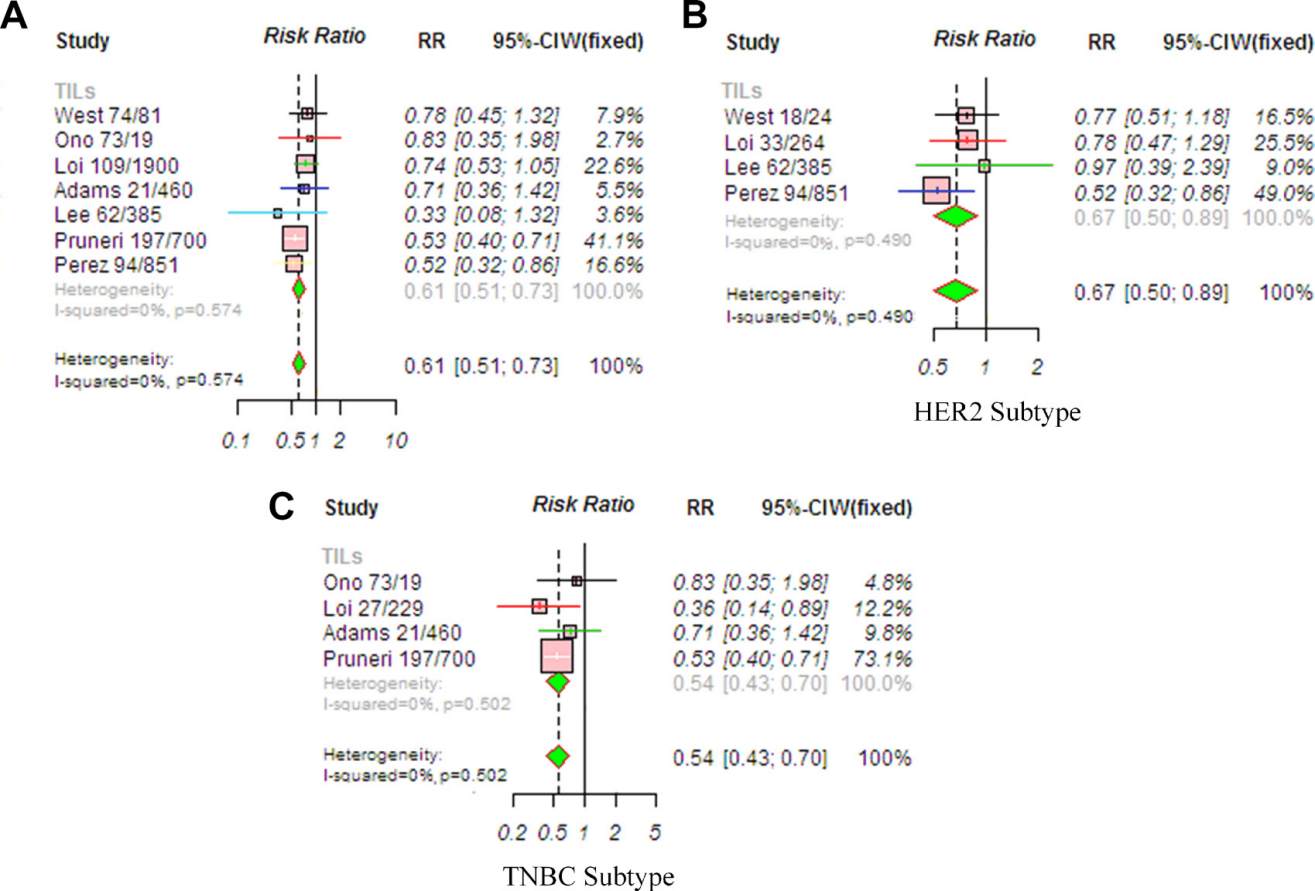
Meta-analysis of relative risk (RR) for the correlation between TIL status and DFS (**A**) Overall population; (**B**) HER2-positive disease; (**C**) TNBC disease. RR values with 95% confidence intervals for survival are associated with high versus low TILs; an RR < 1 represents a lower risk of death or progression. The analysis showed that patients with a high TIL level had significantly improved DFS. Abbreviations: TILs, tumor-infiltrating lymphocyte; TNBC, triple-negative breast cancer; HER2, human epithelial growth factor receptor 2.

The pooled RR for DFS was available in seven studies involving patients with breast cancer. Because the heterogeneity among studies was not significant (*P* < 0.594, *I*^2^ = 0%), the relationship between TIL status and DFS was evaluated using the fixed-effects model. The results showed that a high TIL level was associated with significantly improved DFS (RR, 0.61; 95% CI, 0.51–0.73, *P* < 0.001; Figure 5A). Publication bias was not found in this analysis (Supplementary Table S2). A meta-regression was conducted with the following covariates: markers, detection method, TIL cut-off, and chemotherapy. The results showed that no variable significantly influenced the RR estimate (Supplementary Table S3).

Subgroup analyses of DFS were also performed. Because the heterogeneity among studies was not significant (*P* = 0.490, *I*^2^ = 0%), we used the fixed-effects model. In the HER2-positive subtype, a high TIL level (RR, 0.67; 95% CI, 0.50–0.89, *P* = 0.005) was significantly associated with prolonged DFS (Figure 5B). In addition, the estimated pooled RR showed that a high TIL level was associated with significantly improved DFS (RR, 0.54; 95% CI, 0.43–0.70, *P* < 0.001) in TNBC subtype (Figure 5C). Because the heterogeneity among studies was not significant (*P* = 0.502, *I*^2^ = 0%), we used the fixed-effects model.

### Effects of TILs on overall survival (OS) in patients with breast cancer

**Figure 6:**
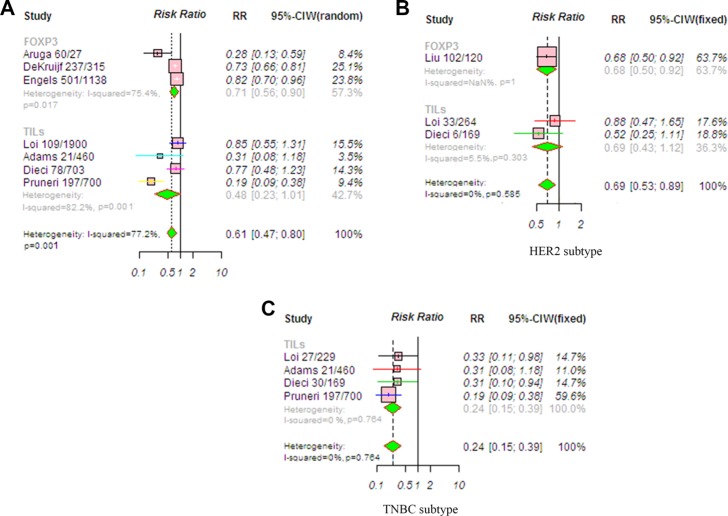
Meta-analysis of relative risk (RR) for the correlation between TIL status and OS (**A**) Overall population; (**B**) HER2-positive disease; (**C**) TNBC. RR values with 95% confidence intervals for survival are associated with high versus low TILs; an RR < 1 represents a lower risk of death or progression. The analysis showed that patients with a high TIL level had significantly improved OS. Abbreviations: TILs, tumor-infiltrating lymphocyte; TNBC, triple-negative breast cancer; HER2, human epithelial growth factor receptor 2.

The pooled RR was used to analyze survival outcomes in patients who were treated with chemotherapy. The random-effects model was applied because heterogeneity across the studies was significant (*P* = 0.001, *I*^2^ = 77.2%). The estimated pooled RR found a significantly longer OS (RR, 0.61; 95% CI, 0.47–0.80, *P* < 0.001) in patients with a high TIL level (Figure 6A). The trim-and-fill analysis revealed that two studies might be missing and that if these were published, the adjusted OR would be 0.76 (95% CI, 0.69–0.82, *P* < 0.001). A meta-regression was conducted with the following covariates: markers, detection method, TIL cut-off, and chemotherapy. The results found that no variable significantly influenced the RR estimate (Supplementary Table S4).

RRs for OS according to TIL status in various subgroups were also evaluated. The fixed-effects model was applied because heterogeneity across studies was not significant (*P* = 0.585, *I*^2^ = 0%). A high TIL level significantly predicted longer OS (RR, 0.69; 95% CI, 0.53–0.89, *P* = 0.005) in the patients with HER2-positive breast cancer (Figure 6B). Moreover, there was a significantly reduced risk of mortality in TNBC patients with high TIL levels (RR, 0.24; 95% CI, 0.15–0.39, *P* < 0.001; Figure 6C). The fixed-effects model was applied because heterogeneity across studies was not significant (*P* = 0.764, *I*^2^ = 0%).

## DISCUSSION

This meta-analysis was based on large pooled clinical cohorts (13,100 cases) and differed substantially from other relevant reports, which only considered a smaller studies and only analyzed pCR. We assessed 23 studies that evaluated the usefulness of TILs in predicting the pCR rate after primary chemotherapy as well as predicting prognosis. Our results indicate that a high TIL level in tumor tissue significantly increased the pCR rate after chemotherapy and significantly improved DFS and OS.

The strongest predictor for pCR that we identified was the CD8 lymphocyte level, but CD4 lymphocyte and Foxp3 cell levels were not predictive. The reasons for these results are unclear, but it is possible that chemotherapy-induced cell death, which releases tumor antigens that can be taken up and processed by antigen-presenting cells (APCs) to CD8+ T cells, leading to the direct and destroy cancer cells by activated CD8+ cells. Interestingly, a comprehensive analysis of inflammatory immune characteristics of breast cancer also indicated a decrease in CD4 lymphocytes and an increase in CD8 lymphocytes. The application of immune modulation to regulating immune system kill cancer cells is an attractive alternative to current therapeutic method.

Breast cancer is a heterogeneous disease and has been sub-grouped into several phenotypically diverse cancers, based on specific molecular features [30]. One report revealed differences in the pCR rate among breast cancer subtypes following chemotherapy [31]. Here, we found that TILs were associated with the clinical parameters of biologically aggressive phenotypes, such as high tumor grade or ER/PR negativity, but they were not associated with HER2 expression. Interestingly, an increase in the number of TILs in patients with HER2-positive and triple-negative breast cancers was associated with a higher rate of pCR as well as longer OS. This result indicates that an elevated immune response in patients with HER2-positive and triple-negative breast cancer is more likely to result in pCR after treatment and that immune factors play a substantial role in long-lasting tumor remission. Currently, the majority of patients with HER2-positive breast cancer are likely to choose chemotherapy plus trastuzumab treatment. This observation may be that HER2-positie tumor cells might interact with the immune system in a more subtle way than do TNBC subtypes. Immune suppressive pathways often activated by TNBC cells, and allow cancer cells escape from host antitumor response. Accordingly, a key question is to shed light on HER2-mediated immune mechanism. Because studies investigating the relationship between survival and changes in subtypes of TILs are limited, large prospective cohort studies are needed.

This meta-analysis has some limitations that need to be addressed in future studies. First, our study was based on individual unadjusted ORs and RRs from published results, not from individual patient data. Individual patient data could provide better accuracy and a more robust estimate of specific associations. However, our conclusions are unlikely to change if the studies were adjusted in multivariate analyses. Second, TIL markers varied in our meta-analysis. Particularly, the TIL marker, cut-off level, and therapeutic details differed, which may have partly influenced the significance of the clinicopathological outcomes in the pCR and survival analyses. Moreover, the included studies have used two detection methods to determine TIL levels and different quantification criteria. Therefore, validation of the reliable value of TILs should be conducted based on a homogeneous group of patients through large prospective studies. In addition, to minimize the effect of bias, we tried to evaluate all relevant issues and data, but it is unavoidable that some data could still be missing. It is possible to reflect negative results that may reduce the reliable value of TIL levels. However, we performed the trim-and-fill analysis and found that the association between TIL levels and pCR and improved survival was still significant, even if these missing studies were published.

In summary, our results strongly suggest that strong lymphocytic infiltration, mainly by CD8 lymphocytes, is associated with improved pCR, particularly in patients with TNBC. Moreover, a high TIL level was strongly correlated with prolonged survival. The clinical advantage of increased numbers of TILs provides an impetus for future research to integrate novel immunotherapy with conventional therapy, and additional studies should focus on immune regulation mechanism in different breast cancer subtypes and understand anticancer immune response in HER2-positive breast cancer and TNBC.

## MATERIALS AND METHODS

### Literature search

We searched the literature using the PubMed database (latest update: January 2016) and combinations of the following search algorithms: lymphocytes, immune responses or chemotherapy, breast or mammary, and cancer or carcinoma or tumor or malignancy. Because some trials concerning the immune system-influenced response to chemotherapy and breast cancer prognosis may not be published, we searched for relevant abstracts published in major international proceedings (The American Society of Clinical Oncology). The reference lists of relevant studies were also searched manually.

### Eligibility criteria

All studies were screened based on the following inclusion criteria: (a) patients diagnosed with breast cancer; (b) pCR or prognostic role of TILs provided; (c) adequate data provided for estimating the odds ratio (OR) or relative risk (RR) for pCR or survival outcome; (d) original article; and (e) written in English. When two or more studies were published by the same authors, the better-quality paper was included. Studies that only examined the expression of TILs or a lower limit of the number of patients were excluded. Letters to the editor, reviews, and comments were also excluded.

### Data extraction

To ensure the quality of the data, two authors (WK and XD) extracted the data independently from all eligible studies. Uncertainties were resolved through discussion. The following data were extracted directly from the publication and Supplemental Materials: first author's name, patient's country, publication year, number of patients, TIL detection method, TIL markers, TIL cut-off value, pCR definition, outcome measures, and median follow-up duration. If survival information was not provided, we calculated the data from the available Kaplan–Meier curves to estimate survival outcome. The relevant information was carefully extracted according to the following three aspects: (1) TIL status was correlated with clinicopathological parameters; (2) TIL status was associated with pCR in breast cancer and subtype, and (3) the TIL value was associated with disease-free survival (DFS) or OS.

The prognostic effects of clinical outcome were pCR, disease-free survival (DFS), and OS, and subgroup analyses were also performed. pCR was defined as the absence of residual invasive cancer cells in breast. DFS was defined as the time after surgery without disease relapse or patient death. OS was defined as the period from surgery until the date of death. Because different studies used different definitions for high and low levels of TILs, we considered either the presence (versus absence), positive (versus negative), or high (versus low) expression and described these as “high” (or “low”) TILs in our analysis. The tumor stage category was determined according to AJCC standard (stage 1–2 vs. stage 3). Hormone receptor positivity was defined as either estrogen receptor (ER) ≥ 1% or progesterone receptor (PR) ≥ 1%, as determined by immunohistochemistry (IHC). HER-2 positivity was defined as either IHC 3+ or FISH positivity. Discrepancies were resolved by discussion with a third reviewer (TZ) until the two original reviewers reached consensus.

We identified each result as an independent data set when more than one marker was used to detect TILs, and when the OR or RR for pCR or survival outcome was reported for different markers. Additional subgroup analyses were performed according to breast cancer subtype (HER2-positive and TNBC). Furthermore, the most frequently reported subsets, including generalized tumor inflammatory infiltrates as well as CD4, CD8, FOXP3 lymphocytes, were assessed.

### Statistical analysis

The pooled OR or RR with a 95% confidence interval (CI) was used to analyze the TIL value in this meta-analysis. It was calculated using fixed-effects or random-effects models, as described by Parmar et al. [32]. Heterogeneity between studies was assessed using Cochran's Q method and the *I*^2^ statistic. The random-effects estimate was presented when the *P*-value was < 0.05. Otherwise, a fixed-effects model estimate was considered. Egger's test and Begg's funnel plot were used to assess possible publication bias. A meta-regression analysis was conducted to estimate the influence of individual studies on the pooled effect. Moreover, we assumed that data are missing at random, and therefore observed study characteristics were used to impute missing data by means of multiple imputations. All statistical analyses were conducted using R-based open-source meta-analysis software (version 2.14.2). The tests were two-sided, and *P*-values < 0.05 were considered significant.

## SUPPLEMENTARY MATERIALS


