# Supplementary material for: Tumor-infiltrating lymphocytes in breast cancer predict the response to chemotherapy and survival outcome: A meta-analysis
Source: Oncotarget. 2016 Jun 13;7(28):44288–98. doi: 10.18632/oncotarget.9988 (PMC5190096; doi:10.18632/oncotarget.9988)
Supplement: Supplementary file 1 [file oncotarget-07-44288-s001.pdf]

Tumor-infiltrating lymphocytes in breast cancer predict the response to chemotherapy and survival outcome: A meta-analysis

Supplementary Materials

Supplementary Table S1: Results of meta-regression analysis exploring the source of heterogeneity with pCR

Covariates	Univariate analysis		
	Coefficient	SE	*P* value
Marker	−0.01	0.12	0.97
Detection method	−0.16	0.15	0.30
Cut-off of TILs	−0.04	0.06	0.52
Chemotherapy	−0.15	0.10	0.14

Supplementary Table S2: Egger's test of funnel plot asymmetry

Clinicopathological parameters	*t* value	df	*P* value
Tumor stage	2.14	11	0.059
HER2 expression	.1.11	7	0.310
ER/PR positive	2.12	11	0.065
Pathological complete response (pCR)	3.17	19	0.005
pCR in HER2-positive subtype	3.63	8	0.007
pCR in TBNC subtype	3.63	8	0.007
Disease-free survival (DFS)	0.53	5	0.621
DFS in HER2-positive subtype	0.52	2	0.657
DFS in TNBC subtype	0.41	2	0.717
Overall survival (OS)	–1.74	5	0.142
OS in HER2-positive subtype	0.11	1	0.928
OS in TNBC subtype	2.21	2	0.052

Supplementary Table S3: Results of meta-regression analysis exploring the source of heterogeneity with DFS

Covariates	Univariate analysis		
	Coefficient	SE	*P* value
Detection method	–0.42	0.23	0.06
Cut-off of TILs	0.21	0.22	0.36
Chemotherapy	0.03	0.25	0.91

Supplementary Table S4: Results of meta-regression analysis exploring the source of heterogeneity with OS

Covariates	Univariate analysis		
	Coefficient	SE	*P* value
Marker	−0.20	0.22	0.47
Detection method	0.12	0.43	0.78
Cut-off of TILs	0.05	0.22	0.83
Chemotherapy	0.10	0.16	0.54
